# Exome sequencing overrides formal genetics: *ASPM* mutations in a case study of apparent X-linked microcephalic intellectual deficit

**DOI:** 10.1111/j.1399-0004.2012.01901.x

**Published:** 2013-04-19

**Authors:** F Ariani, F Mari, S Amitrano, C Di Marco, R Artuso, E Scala, I Meloni, R Della Volpe, A Rossi, H Bokhoven, A Renieri

**Affiliations:** 1 Medical Genetics, University of SienaSiena, Italy; 2 Genetica Medica, Azienda Ospedaliera Universitaria SeneseSiena, Italy; 3 Clinical Neurophysiology, Department of Neurological, Neurosurgical and Behavioral Sciences, University of SienaItaly; 4 Department of Human Genetics, Nijmegen Center for Molecular Life Sciences (NCMLS) & Donders Institute for Brain, Cognition and Behaviour, Radboud University Nijmegen Medical CentreNijmegen, The Netherlands

**Fig. 1:**
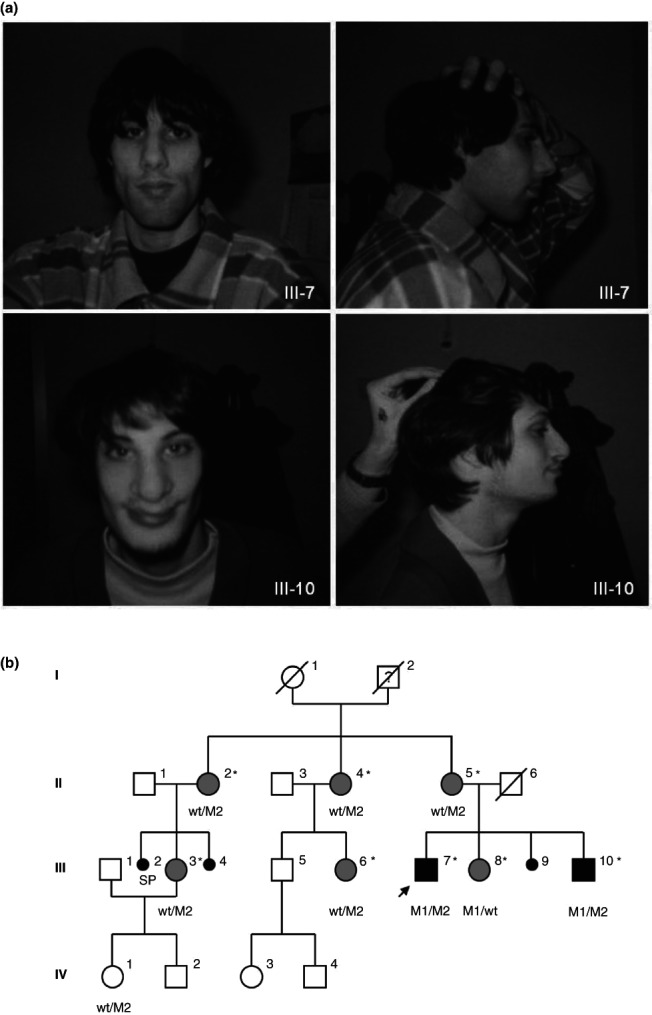
Photographs of the two brothers and the pedigree of the family. (**a**) Patients' pictures at the time of the last examination: III-7 (MR30) at 29 years and III-10 (MR29) at 20 years. Note the receding forehead and squared chin in both brothers. Patient III-7 exhibited hypotelorism. Patient III-10 had a broad nose and a surgical scar for cleft lip/cleft palate. (**b**) Pedigree of the family. Black symbols represent patients with ID and microcephaly, while grey symbols indicate individuals with isolated microcephaly. *: individuals tested for linkage mapping. The genotype at the *ASPM* locus is indicated below tested individuals. M1 = p.E1266X and M2= p.E2605fs. wt = wild-type. II-5 showed an OFC of 48.5 cm (−5.2 SD) and height of 165 cm (50–75° percentile); III-8 OFC of 50.5 cm (−3.4 SD), and height of 167 cm (75° percentile); II-2 OFC of 52.5 cm (−1.6 SD) and height of 170 cm (75–90° percentile); II-4 OFC of 52 cm (−2.1 SD) and height of 165 cm (50–75° percentile); III-3 OFC of 50.5 cm (−3.5 SD) and height of 160 cm (25–50° percentile); III-6 OFC of 52 cm (−2.1 SD) and height of 180 cm (>97° percentile); IV-1 OFC of 52 cm (mean) and height of 120 cm (25–50° percentile). Measurements have been taken in adult age for all subjects except for IV-1, who was 7 years old.

To the Editor:

Ten years ago we evaluated two brothers with intellectual disability (ID). The 29-year-old brother presented with moderate-severe ID with congenital microcephaly [(occipital-frontal circumference (OFC) 42.5 cm, −7.8 SD; height 182 cm, 75°–90° percentile] (Fig. 1a). He was not able to read but was able to write his name. The younger 20-year-old brother exhibited severe ID, congenital microcephaly (OFC 41 cm, −8.7 SD; height 160 cm, 5° percentile) and had a surgically corrected cleft lip and palate (Fig. 1a). He was unable to read or write. Neither patient was able to take care of himself and both exhibited hyperactivity with auto- and hetero-aggressive behavior. Brain MRI of the older brother showed a global reduction in brain size, thin brain stem, normal corpus callosum and temporal pachygiria. Cardiac ultrasound of both patients revealed mild tricuspid valve insufficiency in the older brother and mild tricuspid and pulmonary valve insufficiency in the younger. The mother (II-5) and the 26-year-old sister (III-8) had microcephaly (Fig. 1b). Their father died at 40 years of heart failure. Both the mother and the sister reported females with a small head size in the maternal branch of the pedigree (Fig. 1b). In most of these females microcephaly was directly ascertained. The genetic counseling concluded that there was an X-linked semi-dominant disorder and that there was a recurrence risk for ID of up to 50% for male offspring of the sister (III-8).

After genetic counseling a number of genetic tests were performed. We first tested a panel of genes involved in X-linked ID (*MECP2*, *ARX*, *OPHN1*, *FMR2*, *NLGN3*, *NLGN4*, *PQBP1*, *RSK2*, *AGTR2*) because the samples were enrolled in the Italian X-linked Mental Retardation Project (http://www.biobank.unisi.it/) 1. In the following years, with the advent of array-CGH technology, the samples were first analyzed with X-specific BAC array-CGH and successively with higher resolution arrays (Agilent 105K, Agilent Technologies, Santa Clara, CA and Affymetrix Genome-Wide Human SNP Array 6.0, Affymetrix, Santa Clara, CA); all of these tests were negative. Meanwhile, linkage analysis specific for the X chromosome was performed and four candidate regions segregating in all family members with microcephaly were identified. Bioinformatic analysis indicated *CASK* as a good candidate gene for microcephaly. However, Sanger sequencing of the *CASK* coding region did not reveal any pathogenic mutations. We, therefore, employed “whole exome sequencing” (Illumina platform) to perform an unbiased analysis of the functional portion of the genome in the two affected brothers. We first examined the genes on the X chromosome but did not find any potential mutation for the observed phenotype. Subsequent analysis of the other chromosomes revealed unexpected findings on chromosome 1. The two brothers each carried a frameshift and stop mutation leading to truncations (c.3796G > T, p.E1266X and c.7815_7816del, p.E2605fs) in the *ASPM* gene (abnormal spindle-like microcephaly-associated; MIM#605481). This gene is known to be responsible for autosomal recessive primary microcephaly (MCPH; 2, 3). Segregation analysis performed by traditional sequencing identified the stop mutation in the sister (III-8). The frameshift mutation was identified in the mother (II-5) and in all family members (II-2, II-4, III-3, III-6) with microcephaly, as well as in a family member with normal head size (IV-1; Fig. 1b). *ASPM* is one of the seven genes associated with MCPH, a very rare disorder characterized by reduced head circumference at birth with variable degrees of ID 2. Initially found in consanguineous Pakistani families and later identified in Caucasian MCPH cases, *ASPM* mutations rarely cause microcephaly in the heterozygous state 2, 4, 5.

This case study shows that strategies employing formal genetics could be, in some cases, misleading and that unbiased methods, such as whole exome sequencing, can lead to a rapid genetic diagnosis. In fact, the analysis of the pedigree led us to hypothesize an X-linked semi-dominant inheritance causing microcephaly in females and microcephaly associated with ID in males. In order to test this hypothesis, we investigated genes on the X chromosome by linkage approach and conventional screening methods. At the end of these efforts, we planned to perform the analysis of the entire X chromosome but, since the cost was similar to that of exome sequencing, we chose the latter approach. Exome sequencing data led us directly to the genetic diagnosis and to reconsider our hypothesis based on pedigree analysis, a phenomenon that could be called “reverse formal genetics”. Furthermore, since MCPH is a genetically heterogeneous condition, exome sequencing would still represent the best diagnostic approach, allowing a faster and more cost efficient analysis as compared to traditional techniques. In summary, these results indicate that exome sequencing may supersede “linkage-plus-candidate-gene-sequencing” as an approach to identify relevant pathology-associated genes, even when pedigree analysis is strongly suggestive. Moreover, this case study emphasizes an important clinical point: heterozygous individuals for *ASPM* mutations may manifest microcephaly without serious cognitive dysfunction. Finally, results obtained by exome sequencing were timely because the microcephalic sister (III-8), carrier of the p.E1266X mutation, was actually in the 8th week of a pregnancy and had come to the Unit of Medical Genetics for prenatal counseling. Sequencing data allowed us to define a negligible risk of recurrence, instead of 25% based on our assumption of a semi-dominant X-linked condition.
